# Comparative barrier membrane degradation over time: Pericardium versus dermal membranes

**DOI:** 10.1002/cre2.414

**Published:** 2021-05-05

**Authors:** Fabien Bornert, Valentin Herber, Rebecca Sandgren, Lukasz Witek, Paulo G. Coelho, Benjamin E. Pippenger, Shakeel Shahdad

**Affiliations:** ^1^ Faculty of Dental Surgery, Department of Oral Surgery University of Strasbourg Strasbourg France; ^2^ Department of Dentistry and oral Health, Division of Oral Surgery and Orthodontics Medical University of Graz Graz Austria; ^3^ Biomedical Services Division Lund University Lund Sweden; ^4^ Department of Biomaterials New York University College of Dentistry New York New York USA; ^5^ Department of Biomedical Engineering New York University Tandon School of Engineering Brooklyn New York USA; ^6^ Department of Mechanical and Aerospace Engineering New York University Tandon School of Engineering Brooklyn New York USA; ^7^ Hansjörg Wyss Department of Plastic Surgery New York University School of Medicine New York New York USA; ^8^ Department of Preclinical & Translational Research Institut Straumann AG Basel Switzerland; ^9^ Center for Dental Medicine, Department of Periodontology University of Zurich Zurich Switzerland; ^10^ Institute of Dentistry, Barts and The London School of Medicine and Dentistry Queen Mary University of London London UK

**Keywords:** BioGide, bovine xenograft, collagen membrane, guided bone regeneration, Jason, periodontal defects

## Abstract

**Objective:**

The effectiveness of GBR procedures for the reconstruction of periodontal defects has been well documented. The objective of this investigation was to evaluate the degradation kinetics and biocompatibility of two resorbable collagen membranes in conjunction with a bovine xenograft material.

**Materials and Methods:**

Lower premolars and first molars were extracted from 18 male Yucatan minipigs. After 4 months of healing, standardized semi‐saddle defects were created (12 mm × 8 mm × 8 mm [*l˙̇* × *W˙* × *d*]), with 10 mm between adjacent defects. The defects were filled with a bovine xenograft and covered with a either the bilayer collagen membrane (control) or the porcine pericardium‐derived collagen membrane (test). Histological analysis was performed after 4, 8, and 12 weeks of healing and the amount of residual membrane evaluated. Non‐inferiority was calculated using the Brunner‐Langer mixed regression model.

**Results:**

Histological analysis indicated the presence of residual membrane in both groups at all time points, with significant degradation noted in both groups at 12 weeks compared to 4 weeks (*p* = .017). No significant difference in ranked residual membrane scores between the control and test membranes was detected at any time point.

**Conclusions:**

The pericardium‐derived membrane was shown to be statistically non‐inferior to the control membrane with respect to resorption kinetics and barrier function when utilized for guided bone regeneration in semi‐saddle defects in minipigs. Further evaluation is necessary in the clinical setting.

## 1 INTRODUCTION

The treatment protocol of isolating a defect site in an effort to improve tissue healing and guided regeneration of native tissue utilizing a mechanical barrier has been extensively explored in reconstructive surgery, prior to the development of dental implants in the 1950s (Bassett, Campbell, Girado, Rossi, & Seymour, 1956; Bornstein, Heynen, Bosshardt, & Buser, 2009; Hurley, Stinchfield, Bassett, & Lyon, 1959). However, in discipline of oral and maxillofacial reconstruction, deficiency of bone remains a major issue, originating from systemic and periodontal diseases, tooth loss, trauma and/or pathology (Elgali, Omar, Dahlin, & Thomsen, 2017). As a result, physiological and progressive structural rearrangement of the hard and soft tissues often results in bone atrophy (Araujo & Lindhe, 2005; Bartee, 2001; Sclar, 2004; Trombelli et al., 2008). In an effort to improve bone response and predictably regenerate lost tissue, providing an anatomically analogous ridge contour for biomechanically favorable and prosthetically‐driven implant placement, guided bone regeneration (GBR) procedures have been indicated (Benic & Hammerle, 2014; Dimitriou, Mataliotakis, Calori, & Giannoudis, 2012; Elgali et al., 2017; Hammerle & Jung, 2003). The concept of GBR depends on membrane‐based barrier protection of a boney defect void filled with bone grafting material, allowing osteoprogenitor cells to repopulate the defect site by inhibiting the entry of rapidly proliferating non‐osteogenic tissues (Benic & Hammerle, 2014; Dimitriou et al., 2012; Elgali et al., 2017; Hammerle & Jung, 2003). It has been reported that up to 40% of implant treatments require barrier protection procedures (Bornstein, Halbritter, Harnisch, Weber, & Buser, 2008). Moreover, findings in recent literature have demonstrated similar survival rates (90–100%) for implants placed in GBR augmented sites relative to nonaugmented sites after at least 1 year in function (Clementini, Morlupi, Canullo, Agrestini, & Barlattani, 2012; Moraschini, Poubel, Ferreira, & Barboza Edos, 2015).

In an effort to improve bone healing kinetics, the use of physical barriers associated with augmentation procedures has been proposed (Bornstein et al., 2009; Dahlin, Linde, Gottlow, & Nyman, 1988; Elgali et al., 2017; Simion, Scarano, Gionso, & Piattelli, 1996). The utilization of membranes relies on the biological principle of compartmentalized healing, preventing the migration of unwanted cells, sustaining the blood clot/graft in place, therefore allowing bone‐forming cells to reconstruct lost tissue in a restrained environment (Dahlin et al., 1988; Dimitriou et al., 2012; Elgali et al., 2017; Linde, Alberius, Dahlin, Bjurstam, & Sundin, 1993; Simion et al., 1996). The ideal characteristics of GBR membranes are based on four fundamental principles: (a) biocompatibility that allows tissue integration without eliciting inflammatory responses; (b) cellular occlusivity to avoid defect invasion by epithelial and connective tissue cells, which have a faster turnover rate relative to bone tissue; (c) suitable degradation profile to inversely match the rate of new tissue formation; and (d) adequate mechanical and physical properties to allow manageability along with rigidity for defect space maintenance, avoiding membrane collapse and allowing cell migration from the surrounding bone tissue (Elgali et al., 2017; Rakhmatia, Ayukawa, Furuhashi, & Koyano, 2013).

A wide variety of synthetic and naturally‐derived membranes, both resorbable and nonresorbable, are commercially available (Elgali et al., 2017). Currently, the shift from non‐resorbable to bioresorbable membranes, using naturally‐derived materials and/or employing principles of tissue engineering, has become a significant trend in modern GBR procedures (Bottino et al., 2012; Sheikh et al., 2017). Collagen‐membranes have received major attention for biomedical applications due to numerous desirable biological properties (Liu, Yang, al‐Shaikh, & Lane, 1995). In addition to defect space maintenance, collagen‐based membranes demonstrate outstanding hemostatic, chemostatic and cell adhesive characteristics (Behring, Junker, Walboomers, Chessnut, & Jansen, 2008; Bunyaratavej & Wang, 2001; Locci et al., 1997; Postlethwaite, Seyer, & Kang, 1978; Yaffe, Ehrlich, & Shoshan, 1984). Previous reports have indicated that such membranes have the capacity to attract and activate an increased number of gingival fibroblast cells, periodontal ligament fibroblast cells, and osteoblasts relative to other membranes (Behring et al., 2008; Bunyaratavej & Wang, 2001; Locci et al., 1997; Postlethwaite et al., 1978; Yaffe et al., 1984). In contrast, the primary disadvantages of collagen‐based membranes are their lack of rigidity and fast degradation kinetics by endogenous collagenases, so that the barrier function may not remain long enough for tissue regeneration (Bottino et al., 2012; Bunyaratavej & Wang, 2001; Elgali et al., 2017). Different methods of chemical cross‐linking have been used to improve the mechanical properties and collagen matrix stability, thus slowing their degradation rate; however, such processes have been shown to impair bone‐forming cell response and tissue integration (Bottino et al., 2012; Bunyaratavej & Wang, 2001; Elgali et al., 2017; Speer, Chvapil, Eskelson, & Ulreich, 1980). Therefore, improvements of collagen membrane structure and thickness which depend on the collagen source, extraction method and manufacturing processes have been conducted in order to surpass such drawbacks (Bottino et al., 2012).

There is currently an extensive literature evaluating the effectiveness of GBR procedures using xenografts and collagen‐based membranes to reconstruct a variety boney defects (Araujo & Lindhe, 2005; Behring et al., 2008; Benic & Hammerle, 2014; Bottino et al., 2012; Cardaropoli, Araujo, Hayacibara, Sukekava, & Lindhe, 2005; Clementini et al., 2012; Coelho et al., 2012; Dau et al., 2016; Elgali et al., 2017; Tovar et al., 2014). The current in vivo experimental study histomorphometrically evaluated two commercially available porcine‐derived collagen membranes (a dermal‐derived bilayer collagen membrane (control) and a pericardium‐derived membrane (test)) in minipig mandible semi‐saddle defects in terms of resorption kinetics and biocompatibility according to the ISO 10993‐6 guideline.

## 2 MATERIALS AND METHODS

### 2.1 Animal model

A total of 18 adult male Yucatan minipigs aged 20–24 months and approximately 70 kg in weight at the time of the first surgery were acquired for this study. The study was performed in accordance with the Institutional Animal Care and Use Committee (IACUC) and under ethical approval number 16‐BP‐002/Cerabone (Barton's West End Facilities, NJ). The study was conducted under GLP‐compliant conditions and in accordance with ISO 10993‐6 “Biological evaluation of medical devices – Part 6: Tests for local effects after implantation guideline and recommendations” and reported according to the ARRIVE (Animal Research: Reporting of In Vivo Experiments) guidelines regarding all relevant items.

The animals were kept in pens, in cohorts of three and given 1 week to acclimatize to the environmental conditions prior to surgery. They were fed a standard soft food diet (Special Diet Services [SDS], Witham, UK #801586).

### 2.2 Surgical procedures

Prior to both surgical interventions the animals were fasted overnight to prevent vomiting due to the general anesthesia. On the day of the surgery the animals were anesthetized with an intramuscular injection of a 3:10 mixture of midazolam and ketamine HCl (22 mg/kg). During surgery anesthesia was maintained with isoflurane (2–5%) administered via an intubation tube. Additional local anesthesia (Xylocain Dental adrenalin 20 mg/ml + 12.5 mg/ml) was given to reduce the dosage of the systemic anesthetic and to reduce the bleeding during surgery. Post‐operative analgesia was provided by intramuscular injections of buprenorphine (0.05 mg/kg) and banamine (0.1 mg/kg) as required. To prevent post‐operative infections antibiotics (penicillin/streptomycin, 8–10 mg/kg and cephalexin, 3–5 mg/kg) were given intramuscularly for 3 days after surgery.

**FIGURE 1:**
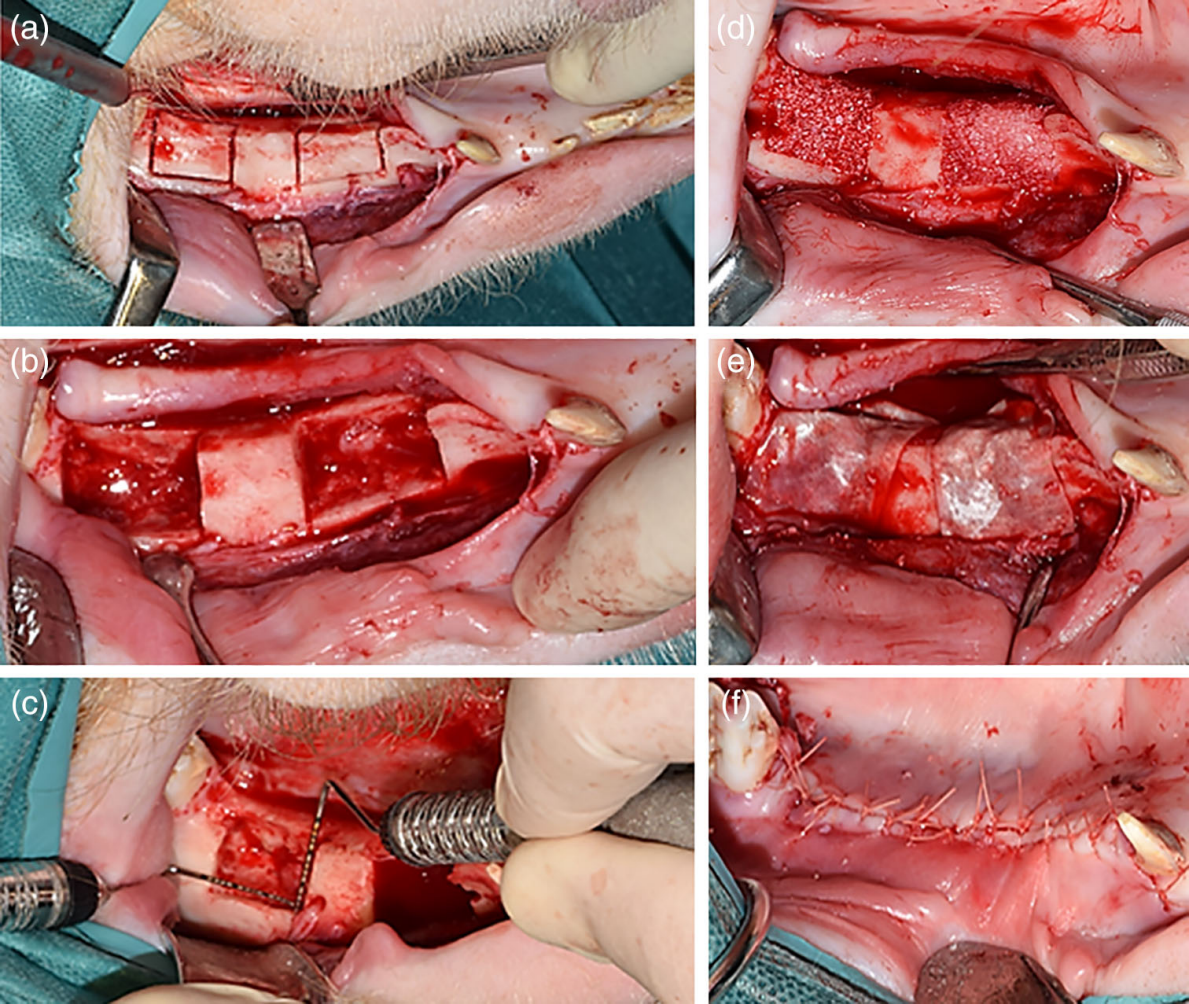
(a), (b) and (c): Creation of semi‐saddle defects in the mandible 4 months after tooth extraction (length = 12 mm, width = 8 mm and depth = 8 mm) with 10 mm between adjacent defects. (c) Defects filled with bovine xenograft (BioOss) granules, 1–2 mm. (e) Defects covered with the collagen membranes (control/BioGide or test/Jason). (f) Flaps closed and sutured

In the first surgery, lower premolars (P2‐P4) and first molar (M1) were carefully extracted bilaterally. Absence of root remnants was confirmed with digital radiographs. The soft tissue was closed using standard surgical technique and the defects were allowed to heal for 4 months, after which four standardized semi‐saddle defects with a defined bucco‐lingual and mesio‐distal size of 10 × 15 mm (two per hemi‐mandible) were created (Figure 1) using the techniques described by Thoma et al. (2012). A mid‐crestal incision was made and a full‐thickness flap raised to expose the mandibular bone between M1 and the canine. The edentulous ridge was then flattened to a width of at least 10 mm under constant saline irrigation. The semi‐saddle defects (length 12 mm, width 8 mm and depth 8 mm) were created with a piezoelectric saw (Mectron, Carasco, Italy), with a distance of 10 mm between two adjacent defects. Two of the defects were then filled with a widely used and examined bovine xenograft (BioOss granules, 1–2 mm, Geistlich [Wolhusen, CH]) and covered with the respective collagen membranes Bio‐Gide (Geistlich (Wolhusen, CH) (control)) or Jason (Botiss (Berlin, DE) (test)) as per manufacturer's instructions. Each membrane was held in place with two titanium pins at the buccal wall corners of the defect. No randomization or permutation of the groups was performed due to the large size of the defects. The remaining two defects were either filled with another bovine xenograft (Cerabone granules, 1–2 mm, Botiss (Berlin, DE)) or left empty, respectively. These defects were covered with the Jason membrane and are subject of a separate manuscript, which investigates the bone formation as a function of bone graft materials and membrane types. The flaps were closed using traditional surgical technique.

### 2.3 Healing periods and sacrifice

The 18 operated animals were split into three groups (*N* = 6/time in vivo) according to different healing times to evaluate healing and resorption kinetics. Therefore, animals were sacrificed according to IACUC protocol after 4, 8 and 12 weeks of healing by inducing cardiac arrest with an intra‐cardiac injection of a 20% solution of pentobarbital. After sacrifice the mandibles were retrieved for histological processing and fixed by immersion in formalin (4% formaldehyde solution) for 2 weeks including repeated change of formalin every second day.

### 2.4 Histologic preparation and analysis

**FIGURE 2:**
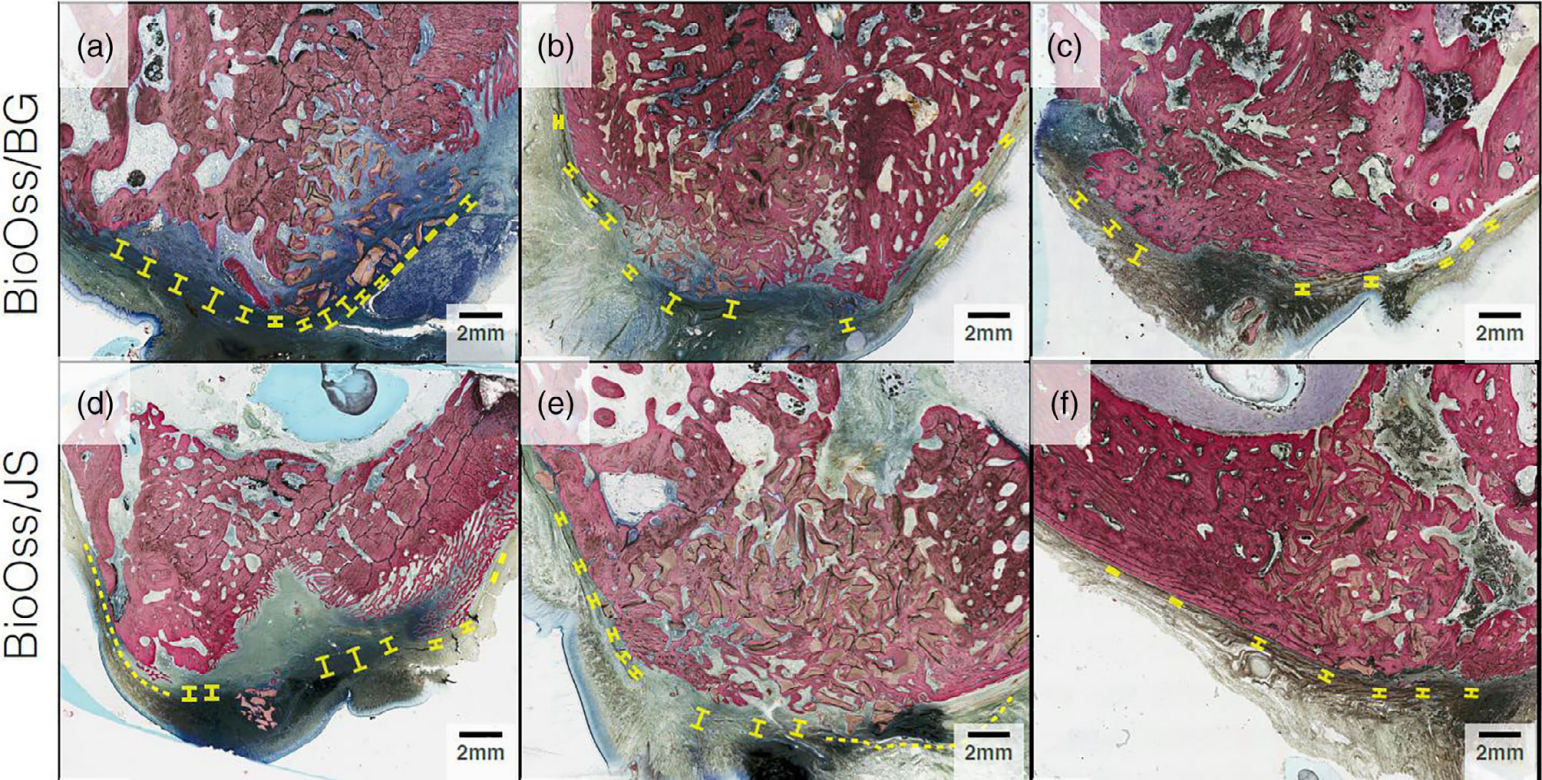
Histological micrographs of the BioOss/control and BioOss/test groups showing decreased membrane thickness due to membrane degradation over time. Left to right columns represent time in 4‐, 8‐ and 12‐week intervals

After fixation the bone blocks were gradually dehydrated in a series of ethanol solutions ranging from 70 to 100%. Following dehydration, the samples were embedded in a methacrylate‐based resin (Technovit 9100; Heraeus Kulzer, Wehrheim, Germany). The blocks were then cut in bucco‐palatal direction into slices approximately 300 μm thick with a precision diamond saw (Isomet 2000; Buehler, Lake Bluff, IL). The slices were then glued to acrylic slides using an acrylate‐based glue and were allowed to set for 24 hr before grinding and polishing. The sections were then reduced to a final thickness of approximately 100 μm by means of a series of SiC abrasive papers (400, 600, 800, 1200, and 2400) in a grinding/polishing machine (Metaserv 3000, Buehler, Lake Bluff, IL) under constant water irrigation. Subsequently, the samples were stained with Stevenel's Blue and Van Giesons's Picro Fuschin (SVG) stains and digitally scanned via an automated slide scanning system and specialized computer software (Aperio Technologies, Vista, CA). The amount of residual membrane presence was ranked by visual observation through the bucco‐lingual cross‐section according to a previously described method (Tovar et al., 2019). In brief, the temporal evolution of barrier function was indirectly assed by the presence of residual membrane. The evaluation was performed by marking any loci along the perimeter of the augmented defect site (yellow brackets and dashes in Figure 2) at which a laminar fibrillary structure could be visually identified that was likely to be associated to one of the used membranes. The presence of membrane was evaluated visually and ranked on a scale from 0 to 5, where 0 denoted a total absence of membrane structures and 5 constituted entire coverage of the surgical site with visible presence of the membrane. The histological evaluation was performed by a blinded, trained observer at the Department of Biomaterials, New York University College of Dentistry.

### 2.5 Biocompatibility

Biocompatibility was assessed according to the ISO 10993‐6 guidelines. Briefly, the inflammatory infiltrate was scored using a standardized 0‐4‐point scoring system (not present—packed/sheets/severe) from the ISO‐norm. Polymorphonuclear cells, lymphocytes, plasma cells, macrophages, giant cells and the presence of necrosis were all included in the analysis. The entire image was analyzed per implant per group and values per category were averaged. Determination of overall irritant or not was made in a semi‐quantitative fashion based on the acceptable deltas found in the ISO‐norm reference document.

### 2.6 Statistical analysis

Equality of distinguishable variances in the ranking of the residual membrane data was calculated using Levene's test, and the data were analyzed using the Kruskal‐Wallis nonparametric one‐way analysis of variance. Data were presented as a function of median and quartiles. Stratified mixed linear regression models were performed using the nonparametric Brunner‐Langer method (Brunner, Domhof, & Langer, 2002) to examine the association between outcome and type of membrane, including the type of membrane as a fixed effect and the animal as a random effect. Non‐inferiority was also evaluated using the Brunner‐Langer mixed linear regression model, calculating the difference between adjusted means of the two membranes and the two‐tailed 90% confidence interval.

For extrapolating the time needed to reach a zero residual membrane ranking, a linear relationship of membrane degradation over time was assumed (based on the raw data of the 3 timepoints included in the present study). A linear regression was first performed on these raw data points to ensure a linear fit was indeed appropriate (the *r* ^2^ values as a goodness of fit determination was used as a reference). The original raw data was then interpolated (GraphPad Prism software, v 8.0) and the resultant data points were graphed on an XY table. The graphed linear line was then extended using the slope formula of the line to determine the *y*‐intercept (time in weeks of zero residual membrane ranking). This was performed for both membranes to determine their comparative theoretical full resorption time.

## 3 RESULTS

Surgical interventions demonstrated no complications regarding procedures, postoperative infections and/or other clinical concerns. Additionally, following euthanasia, sharp dissection of the mandible defects did not reveal any clinical signs of inflammation and/or infection throughout the different in vivo healing periods.

As indicated by the yellow dashed lines and brackets, which was used to mark the visually detectable laminar fibrous structures associated to residual membrane remnants the evaluation of the histological micrographs showed the presence of residual membrane at all time points. Further the diameter of the zones (size of brackets) containing laminar fibrous tissue was seen to be reduced after 12 weeks, which was associated to membrane degradation and soft tissue remodeling, but no clinically significant differences were observed between the two membranes (Figure 2).

Evaluation of data as a function of experimental groups yielded no significant difference between membrane types, irrespective of the graft material (*p* > .093). A significant difference between membrane type was observed when data were collapsed over graft material, with control presenting lower degradation compared to test membranes (*p* = .048).

**FIGURE 3:**
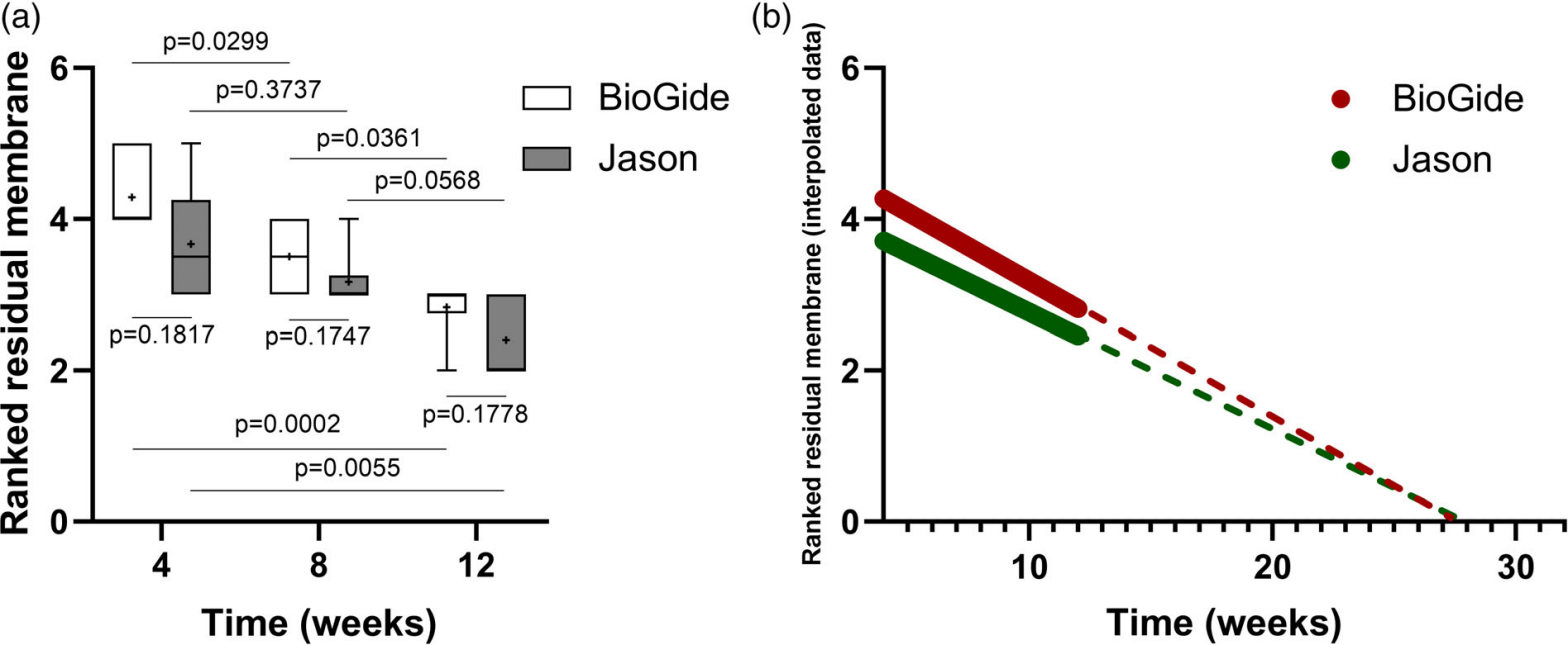
(a) Ranked residual membrane scores for the control and test membrane groups at 4, 8, and 12 weeks. (b) Extrapolation of total membrane degradation, based on interpolated raw data

Ranked residual membrane scores are presented in Figure 3a. Scores for control membranes were significantly different between 4 and 8 weeks (*p* = .029), between 4 and 12 weeks (*p* < .001), and between 8 and 12 weeks (*p* = .036). Scores for test membranes were significantly different between 4 and 12 weeks (*p* = .0055), but not between 4 and 8 weeks (*p* = .374) or between 8 and 12 weeks (*p* = .057). There were no significant differences in ranked residual membrane scores between the control and test membranes at any time point (4 weeks, *p* = .182; 8 weeks, *p* = .175; 12 weeks, *p* = .178). Ranked residual membrane as a function of time demonstrated significant membrane degradation in both groups after 12 weeks compared to 4 weeks (*p* = .017), with no significant difference between 4 and 8 weeks (*p* = .147) (Figure 3a).

Extrapolation of the interpolated ranked residual membrane scores data resulted in best fit lines crossing the *x*‐axis (time in weeks) at comparable times (27.46 weeks for control and 27.59 weeks for test membranes) (Figure 3b). Based on the 95% Confidence Intervals of the *x*‐intercept predictions, test membranes showed a markedly higher variation, stemming from the higher *SD* present in the raw data (control: 21.94 to 39.62 weeks; test: 19.87 to 59.64 weeks). This was also reflected in the different values obtained for the 2 groups' sum of squares (control: 3.776, test: 5.435). However, both values were acceptably low, thus demonstrating the original data did not vary considerably from the mean value.

As illustrated by the values in Table 1 biocompatibility testing confirmed that both membranes were safe and not irritant; no local test item‐related findings were observed. Slightly higher scores were recorded for BioOss/test membrane compared to BioOss/control membrane after 4 and 8 weeks, which was mainly associated to a higher score for lymphocytes. According to the evaluation per guidance ISO‐10993‐6 a slight reaction was observed for the test and control samples at the 4 weeks' time point and for the test samples at the 8 weeks' time point. Minimal or no reaction was observed for the control samples at the 8 weeks' time point and for both samples at the 12 weeks' time point.

**TABLE 1:** Summarized biocompatibility scores comparing test (Jason) and control (Bio‐Gide) membranes according to IOS 10993–6:2007 (E)

	4 weeks	8 weeks	12 weeks
Control (Bio‐Gide)	4.4	0.7	1.0
Test (Jason)	6.6	3.7	0.9

## 4 DISCUSSION

The results of this investigation showed that the degradation and biocompatibility of the test collagen membrane Jason was comparable to the ones of the bilayer collagen test membrane Bio‐Gide when used in combination with a bovine xenograft for the regeneration of bone in semi‐saddle defects in minipigs. Barrier function was similar between the two membranes and remains intact up to 12 weeks in vivo; substantial degradation occurred between 8 and 12 weeks, allowing the bone healing process to take place. The test membrane was statistically noninferior to the control membrane.

This manuscript particularly aimed to analyze and compare membrane function, that is, stabilization of the graft material in the defect and the provision of a physical barrier that inhibits the ingrowth of undesired connective tissue into the graft materials without eliciting any significant inflammatory foreign body reactions (Rothamel et al., 2012). The test system consisted of a previously reported standardized porcine semi‐saddle defect of defined size and shape (Thoma et al., 2012). As part of the study design sample types were not randomized or permutated between the mesio‐distal positions of the defects implying that possible effects of position on membrane degradation were not taken into account. This study further used a previously described semi quantitative method for the evaluation of barrier function based on the amount of residual detectable membrane. Specifically, this latter property was evaluated by rating the visually detectable amount of laminar fibrous tissue in the histological cross sections which was associated to the presence of remaining membranes on a scale from 0 to 5 (Tovar et al., 2019). These aspects might need to be considered for the correct interpretation of the here presented study results.

An investigation into the mechanical properties of three different collagen membranes (test/Jason, control/Bio‐Gide and Collprotect [botiss GmbH]), including quasi‐static, time‐dependent and functional testing, rendered test membranes to be the thinnest membrane (0.20 mm) and control membranes the thickest (0.44 mm) (Ortolani et al., 2015). The test membrane also yielded a lower surface density (40 g/m^2^ compared to 140 g/m^2^ for the control membrane) and higher maximum tensile stress (13.0 MPa compared to 4.8 MPa for the control membrane), as well as a significantly higher elastic modulus (179.9 MPa compared to 15.7 MPa for control). More recent investigations have shown that periodontal regeneration can be influenced by the properties of the material used for GBR. For example, an in vitro study compared the proliferation and adhesion of gingival fibroblasts on three different collagen membranes, test/Jason, BioMend and Regen. Cells were cultured on the membranes and cell survival assessed after 1, 2, 3, and 7 days. The results presented a significant increase in cell survival on the test and Regen membranes after 7 days compared to the BioMend membrane, and cell adhesion was also better on the test and Regen membranes (Talebi Ardakani, Hajizadeh, & Yadegari, 2018). More recently, cell viability, morphology and adhesion of gingival fibroblasts and MG‐63 osteoblast‐like cells on test/Jason, CenoMembrane (SOUQ.DENTAL, Jeddah, Saudi Arabia) and TXT‐200 (Osteogenics, Lubbock, TX) membranes was investigated, where cells were cultured on the aforementioned membranes using platelet‐rich plasma (PRP) or 0.5 or 10% fetal bovine serum (FBS). At 10% FBS, the test membrane showed the highest adhesion for both cell types after 1 and 3 days. The highest amount of cell viability was also observed with the test membrane (Vahabi, Yadegary, & Karamshahi, 2019). Rothamel et al have qualitatively compared the temporal resorption of a different porcine pericardium membrane to the control membrane Bio‐Gide in a dog model and have reported a slightly faster degradation (4 to 8 weeks) of the control membrane compared to the Pericardium membrane (8 to 12 weeks)(Rothamel et al., 2012). The authors have also reported on a comparable tissue integration, comparable underlying bone formation and similar immune response of both types of membranes. Interestingly and by contrast to these results our study displayed a comparable degradation pattern of the membranes but a slightly higher biocompatibility score for the test membrane compared to the control membrane. This resulted in a slightly different grading of the tissue reaction after 8 weeks for the test membrane compared to the control membrane that was associated to a higher frequency of lymphocytes at the implantation sites of the test items. It remains speculative at this point whether the faster than expected degradation of the test membrane might have been causally related to this slightly more pronounced immune response.

Comparable bone regeneration with a combination of the test/Jason membrane and β‐tricalcium phosphate (Ceros TCP) and a combination of the control/Bio‐Gide membrane and Bio‐Oss has been seen in a clinical study evaluating horizontal bone regeneration. The study involved 50 patients with horizontal osseous defects, randomized to receive either test + Ceros TCP (25 patients with 29 implants) or control + Bio‐Oss (25 patients, 32 implants). After 12 months post‐surgery (6 months after implant loading), there were no significant differences in vertical bone gain (estimated difference −0.15 mm; *p* = .550) or horizontal bone gain (estimated difference −0.27 mm, *p* = .385). Similarly, no differences were observed between the groups for complete defect filling. Additionally, there was no statistical differences between the groups for post‐operative pain immediately after surgery or after 1 and 2 weeks. A slight difference in radiographic peri‐implant bone loss was observed (0.245 mm, *p* = .046), in favor of the test + Ceros TCP group (Merli et al., 2015). After 3 years, radiographic peri‐implant bone loss was 1.02 mm in the test + Ceros TCP group compared to the 1.61 mm in the control + Bio‐Oss group (*p* = .31), and there were no significant differences between the groups for functional and esthetic satisfaction or pink esthetic score (Merli et al., 2015).

The present study suggested that the barrier effect of the here studied test membrane Jason is comparable to that of more common membrane types (i.e., the present control membrane; BioGide), and the amount of residual membrane was similar, despite the test membrane initially being thinner in nature. Barrier function remained intact after 12 weeks. The resorption kinetics, combined with the cell adhesion properties shown in previous investigations, therefore facilitates the bone healing process. The physical properties such as the low surface density and high tensile strength may also contribute to the effectiveness of the test membrane. Other properties such as the porosity of the membrane appear to have a substantial contribution to the success of GBR, but the precise role has yet to be elucidated. Research in this field is therefore actively addressing both the mechanical and the bioactive properties of such membranes in order to optimize the use of these materials in clinical practice.

## CONFLICT OF INTEREST

The authors declare no conflict of interest.

## CLINICAL SIGNIFICANCE

Guided bone regeneration procedures remain one of the most common practices in implant dentistry today. The success of the technique is reliant upon the performance of the overlying barrier membrane. Beyond its handling characteristics, the membrane must primarily serve to block the ingrowth of gingival cells into the covered bone defect. To fulfill this obligation, two intrinsic properties must be met: a sufficiently slow degradation profile and a robust structure able to withstand the clinical conditions necessary for placement and subsequent healing. Pericardium barrier membranes have recently been introduced as promising candidates that would not only fulfill the requirements, but also offer an accessible membrane technology to a broad range of clinicians.

## Data Availability

The data that support the findings of this study are available from the corresponding author upon reasonable request.
